# Genetic Characterization of the Tick-Borne Orbiviruses

**DOI:** 10.3390/v7052185

**Published:** 2015-04-28

**Authors:** Manjunatha N. Belaganahalli, Sushila Maan, Narender S. Maan, Joe Brownlie, Robert Tesh, Houssam Attoui, Peter P. C. Mertens

**Affiliations:** 1 Vector-borne Viral Diseases Programme, The Pirbright Institute, Ash Road, Pirbright, Woking, Surrey GU24 0NF, UK; E-Mails: manjunatha.belaganahalli@pirbright.ac.uk (M.N.B.); houssam.attoui@pirbright.ac.uk (H.A.); 2 Department of Animal Biotechnology, LLR University of Veterinary and Animal Sciences, Hisar 125 004, Haryana, India; E-Mail: narendermaan108@gmail.com; 3 Department of Pathology and Infectious Diseases, Royal Veterinary College, Hawkshead Lane, North Mymms, Hatfield, Herts AL9 7TA, UK; E-Mail: jbrownlie@rvc.ac.uk; 4 Department of Pathology, University of Texas Medical Branch, 301 University Boulevard, Galveston, TX 77555–0609, USA; E-Mail: rtesh@utmb.edu

**Keywords:** Chenuda virus, Chobar Gorge virus, Wad Medani virus, Great Island virus, Kemorovo virus, *Orbivirus*, *Reoviridae*, full genome, dsRNA virus, sequencing

## Abstract

The International Committee for Taxonomy of Viruses (ICTV) recognizes four species of tick-borne orbiviruses (TBOs): *Chenuda virus*, *Chobar Gorge virus*, *Wad Medani virus* and *Great Island virus* (genus *Orbivirus*, family *Reoviridae*). Nucleotide (nt) and amino acid (aa) sequence comparisons provide a basis for orbivirus detection and classification, however full genome sequence data were only available for the *Great Island virus* species. We report representative genome-sequences for the three other TBO species (virus isolates: Chenuda virus (CNUV); Chobar Gorge virus (CGV) and Wad Medani virus (WMV)). Phylogenetic comparisons show that TBOs cluster separately from insect-borne orbiviruses (IBOs). CNUV, CGV, WMV and GIV share low level aa/nt identities with other orbiviruses, in ‘conserved’ Pol, T2 and T13 proteins/genes, identifying them as four distinct virus-species. The TBO genome segment encoding cell attachment, outer capsid protein 1 (OC1), is approximately half the size of the equivalent segment from insect-borne orbiviruses, helping to explain why tick-borne orbiviruses have a ~1 kb smaller genome.

## 1. Introduction

The orbiviruses are icosahedral, non-enveloped dsRNA viruses belonging to the genus *Orbivirus* within the family *Reoviridae.* The genus currently includes 22 species (representing 22 distinct virus serogroups) that have been recognized by the International Committee for the Taxonomy of Viruses (ICTV) [1]. Recent phylogenetic comparisons of isolates from different *Orbivirus* species, with ‘unclassified’ isolates from the genus, have led to proposals to ICTV for recognition of seven additional species [1,2,3,4,5,6,7,8].

The orbiviruses have a wide host range that collectively includes domestic and wild ruminants, equines, marsupials, sloths, bats, birds and humans [1,9,10,11,12]. They also infect and are transmitted by a range of hematophagus arthropods, including *Culicoides*, phlebotomines (sandflies), mosquitoes and ticks. The tick-borne orbivirus (TBOs) species include *Chenuda virus*, *Chobar Gorge virus*, *Wad Medani virus* and *Great Island virus*. It has been suggested that ‘Kemerovo virus’ (currently a sub-group within *Great Island virus*) could also be recognized as a separate species [13,14]. The genus includes one ‘tick orbivirus’ St Croix River virus (SCRV), as the only member of the distinct and more distantly related species *St Croix River virus*.

The species *Chenuda virus* includes seven serotypes/strains: Chenuda virus (CNUV), Baku virus (BAKUV), Essaouira virus (ESSV), Huacho virus (HUAV), Kala Iris virus (KIRV), Mono Lake virus (MLV) and Sixgun city virus (SCV). CNUV was isolated in 1954 from ticks in Egypt, with serological evidence of infection in birds, camels, pigs, buffalo, dogs, donkeys and rodents [9]; BAKUV was isolated in 1970 in the USSR [9]; and HUAV was isolated in Peru in 1967, while MLV and SCV were isolated in 1966 and 1969, respectively, in the USA. The geographical distribution of ESSV and KIRV has been described in Morocco [15]. The species *Chobar Gorge virus* includes two serotypes/strains: Chobar Gorge virus (CGV) isolated in 1970 from *Ornithodoros spp.* ticks in Nepal; and Fomede virus (FV) isolated in 1978 from a bat in Kindia, Guinea. There is serological evidence for infection of cattle, horses, sheep, buffalo and humans [9]. The species *Wad Medani virus* includes two serotypes: Wad Medani virus (WMV) isolated in 1952 from ticks collected at Wad Medani in Sudan; and Seletar virus (SELV) isolated in 1961 from ticks collected in the Seletar district, Singapore. There is serological evidence for infection of cattle, camel, pigs, buffalo and rodents [1,9,16].

The orbivirus genome consists of ten linear segments of dsRNA (Seg-1 to Seg-10 in order of decreasing molecular weight), which are packaged within a triple layered icosahedral protein capsid [1]. The genome segments encode seven structural (VP1 to VP7) and four non-structural (NS1, NS2, NS3/NS3a, and NS4) proteins [17,18,19,20] (Table 1 and Table 2). In recent years, genome sequence data has steadily become more important for virus identification [5,21,22]. Full genome data and phylogenetic comparisons have supported development of faster and more reliable, virus-species specific and virus-serotype specific diagnostic assays for some *Orbivirus* species, using either conventional or real-time RT-PCR [23,24,25]. Sequence data also provide a basis for molecular epidemiology studies, identifying different topotypes, virus lineages and even the origins of the individual genome segments present within reassortant orbivirus strains [5,13,22,26,27,28,29,30].

Full-genome sequence data are now available for representative/reference strains of the ten established species of the *Culicoides-*borne orbiviruses (CBOs), for one phlebotomine-borne orbivirus (PBO) and four of the six mosquito-borne orbiviruses (MBOs). Although full genome sequence data are also available for SCRV (a tick orbivirus (TO)), the genomes from isolates from only one species of TBOs have previously been fully sequenced (for isolates Great island virus (GIV), Kemerovo virus (KEMV) and Tribec virus (TRBV)). We report full genome sequences for representative isolates of the species *Chenuda virus*, *Chobar Gorge virus* and *Wad Medani virus,* providing a basis for further comparisons to other orbiviruses and the identification of novel TBO isolates and species (see Table 1 and Table 2).

## 2. Results

### 2.1. Virus Propagation and Genomic dsRNA Electropherotype

**Figure 1:**
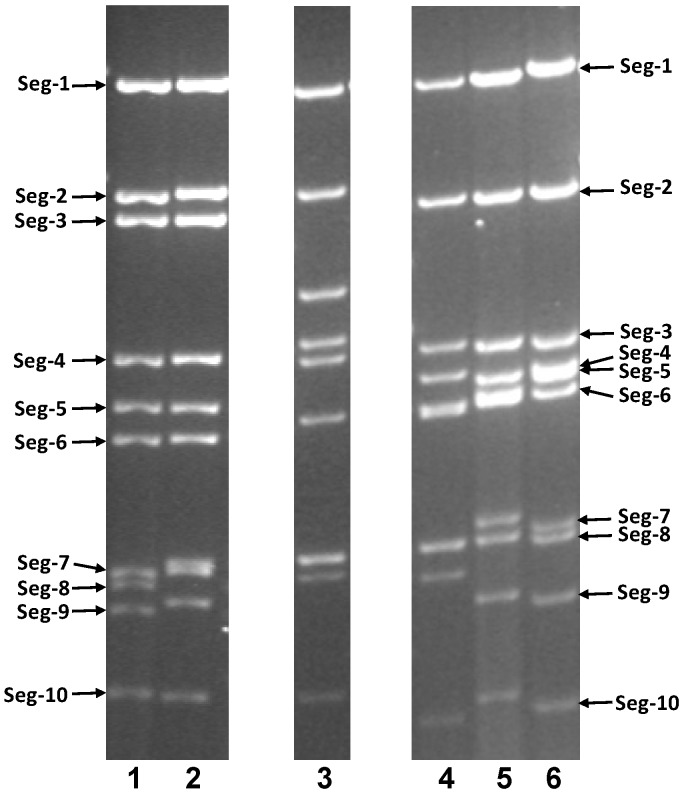
Agarose gel (1%) electrophoretic profile of the dsRNAs of the tick-borne orbivirus isolates Chenuda virus (CNUV), Chobar Gorge virus (CGV) and Wad Medani virus (WMV) along with mosquito-borne and *Culicoides-*borne orbiviruses. Lane 1 = BTV-1w (LIB2007/05); Lane 2 = EHDV-8e (AUS1982/05); Lane 3 = CORV (AUS1960/01); Lane 4 = CGV (NEP1970/01); Lane 5 = CNUV (EGY1954/01); and Lane 6 = WMV (SUD 1952/01).

Isolates of CNUV (EGY1954/01), CGV (NEP1970/01) and WMV (SUD1952/01) were obtained from the Orbivirus Reference Collection (ORC) at The Pirbright Institute [31]. These viruses were used to infect BHK cell monolayers, inducing characteristic cytopathic effects (CPE) at 48 to 72 hours post infection. Genomic dsRNAs purified from these infected cell cultures, were analyzed by 1% agarose gel electrophoresis (AGE) (Figure 1). Each of the tick-borne orbiviruses exhibited an overall 2-4-4 size distribution (2 large, 4 medium and 4 small genome-segments), although considerable variability was observed in the relative migration/sizes of both their ‘medium’ and ‘small’ genome segments (Seg-3 to Seg-6 and Seg-7 to Seg-10) (Figure 1), supporting classification of these isolates within distinct species [1]. The mosquito-borne orbiviruses also show a 2-4-4 distribution (with a different overall size distribution), while the *Culicoides*-borne orbiviruses have a much larger outer capsid protein 1 (OC1) encoding gene, resulting in a 3-3-4 migration pattern.

**Table 1:** Characteristics of dsRNA genome segments and proteins of the Chenuda virus (CNUV), Chobar Gorge virus (CGV) and Wad Medani virus (WMV) viruses.

Virus/Segment	Segment Length (bp)	Protein Encoded	Predicted Protein Length (aa)	Predicted Protein Mass (kDa)	ORFs bp (Including Stop Codon)	5' NCRs	3' NCR	5' Conserved Termini	3' Conserved Termini	% GC Content	Accession Numbers
**CNUV**											
Seg-1	3895	Pol	1285	145.08	11-3868	10	30	5'-GUAAAA	CGAUAC-3'	53.6	KP268794
Seg-2	2787	T2	908	102.62	13-2739	12	51	5'-GUAAAA	UCCUAC-3'	54.7	KP268795
Seg-3	1931	CaP	632	72.14	7-1905	6	29	5'-GUAAAA	GAGUAC-3'	54.9	KP268796
Seg-4	1767	OC1	568	63.34	18-1724	17	46	5'-GUAUAA	ACUUAC-3'	54.3	KP268797
Seg-5	1700	TuP	536	60.8	33-1643	32	60	5'-GUAAAA	UGCUAC-3'	56.3	KP268798
Seg-6	1672	OC2	535	58.92	25-1632	24	43	5'-GUAAAA	GCUUAC-3'	56.5	KP268799
Seg-8	1177	T13	365	40.71	18-1115	17	65	5'-GUAAAA	ACUUAC-3'	57.3	KP268801
Seg-7	1230	ViP	384	42.54	29-1183	28	50	5'-GUAAAA	CGAUAC-3'	57.1	KP268800
Seg-9	1005	Hel	315	33.21	16-963	15	45	5'-GUAAAA	AGCUAC-3'	56.9	KP268802
		NS4	183	21.67	116-667	115	341				
Seg-10	746	NS3	223	23.69	21-692	20	57	5'-GUAAAA	UGAUAC-3'	57.4	KP268803
		NS3a	211	22.3	57-692	56	57				
**Total**	**17910**						**Consensus**	**5'-GUA^A^/_U_AA**	**N^G^/_C/A_NUAC-3`**	55.9	
**CGV**											
Seg-1	3888	Pol	1284	144.64	12-3866	11	25	5'-GUUUA	ACCUAC-3'	52.1	KP268784
Seg-2	2796	T2	909	103.26	15-2744	14	55	5'-GUUUA	AGAUAC-3'	50.6	KP268785
Seg-3	1944	Cap	635	73.1	9-1916	8	31	5'-GUUUA	AGAUAC-3'	52	KP268786
Seg-4	1806	OC1	587	65.88	18-1781	17	28	5'-GUUUA	GGAUAC-3'	50.5	KP268787
Seg-5	1673	TuP	524	59.64	39-1613	38	63	5'-GUUUA	GGAUAC-3'	53.7	KP268788
Seg-6	1644	OC2	535	58.24	17-1624	16	23	5'-GUUUA	AGAUAC-3'	54.9	KP268789
Seg-7	1180	T13	355	39.63	22-1089	21	94	5'-GUUUA	AGAUAC-3'	53.3	KP268790
Seg-8	1167	ViP	369	41.52	20-1129	19	41	5'-GUUUA	AGAUAC-3'	55	KP268791
Seg-9	1093	Hel	346	36.76	15-1055	14	41	5'-GUUUA	AGAUAC-3'	54	KP268792
		NS4	238	28.26	46-762	45	334				
Seg-10	708	NS3	206	23.16	22-642	21	69	5'-GUUUA	AGAUAC-3'	53.8	KP268793
		NS3a*	192	21.64	64-642	63	69				
**Total**	**17899**						**Consensus**	**5'-GUUUA**	**^A^/_G_^G^/_C_^A^/_C_UAC-3'**	53	
**WMV**											
Seg-1	3944	Pol	1303	146.7	11-3922	10	25	5'-GUAUAA	UGCUAC-3'	52.4	KP268804
Seg-2	2791	T2	909	102.37	13-2742	12	52	5'-GUAUAA	AGCUAC-3'	53.4	KP268805
Seg-3	1920	Cap	622	71.21	8-1876	7	47	5'-GUUUAA	GACUAC-3'	52.1	KP268806
Seg-4	1805	OC1	580	65.44	19-1761	18	47	5'-GUAAAA	CGCUAC-3'	53.8	KP268807
Seg-5	1761	TuP	535	60.28	28-1635	27	129	5'-GUAAAA	UGCUAC-3'	54.6	KP268808
Seg-6	1686	OC2	542	58.92	23-1651	22	38	5'-GUUAAA	UGCUAC-3'	53.4	KP268809
Seg-8	1169	T13	354	39.08	19-1083	18	89	5'-GUAAAA	GGCUAC-3'	54.9	KP268811
Seg-7	1207	ViP	376	39.99	26-1156	25	54	5'-GUAAAA	UGCUAC-3'	57	KP268810
Seg-9	997	Hel	313	33.8	14-955	13	45	5'-GUAAAA	UGCUAC-3'	53.2	KP268812
		NS4	189	22.42	102-671	101	329				
Seg-10	729	NS3	219	23.85	15-674	14	58	5'-GUUAAA	UCCUAC-3'	53.5	KP268813
		NS3a	214	23.27	30-674	29	58				
**Total**	**18009**						**Consensus**	**5'-GU^A^/_U_^A^/_U_AA**	**N^G^/_A/C_CUAC-3'**	53.8	
**GIV**											
Seg-1	3897	Pol	1285	146.84	12-3869	10	31	5'-GUAAA	AUCCUAC-3'	55.9	HM543465
Seg-2	2794	T2	908	102.9	19-2745	12	52	5'-GUAAA	AAGAUAC-3'	57.6	HM543466
Seg-3	1936	Cap	635	72.81	6-1913	7	26	5'-GUAAA	AAGCUAC-3'	57.3	HM543467
Seg-4	1722	OC1	551	62.32	18-1673	18	52	5'-GUAAA	AGGAUAC-3'	58.8	HM543469
Seg-5	1731	Tup	531	59.86	41-1636	27	98	5'-GUAAA	AAGAUAC-3'	59	HM543468
Seg-6	1666	OC2	537	59.51	21-1634	22	35	5'-GUAAA	GUCCUAC-3'	58.6	HM543470
Seg-7	1181	T13	357	39.64	18-1091	18	93	5'-GUAAA	AAGAUAC-3'	58.8	HM543471
Seg-8	1172	ViP	359	38.87	46-1125	25	50	5'-GUAAA	AGGAUAC-3'	59.3	HM543472
Seg-9	1056	Hel	321	34.45	55-1020	13	39	5'-GUAAA	AAGGUAC-3'	58.3	HM543473
		NS4	190	22.52	176-748	175	311				
Seg-10	703	NS3	171	19.4	146-661	145	45	5'-GUAAA	AGGAUAC-3'	57.6	HM543474
		NS3a	149	16.99	212-661	211	45				
**Total**	**17858**						**Consensus**	**5'-GUAAA**	**……….UAC-3'**	58.1	
**KEMV**											
Seg-1	3896	Pol	1285	146.01	12-3868	11	31	5'-GUAAAA	AGGAUAC-3'	55.3	HQ266591
Seg-2	2792	T2	908	102.74	19-2745	18	50	5'-GUAAAA	AGGAUAC-3'	57.1	HQ266592
Seg-3	1934	Cap	632	72.4	6-1904	5	33	5'-GUAAAA	AACUUAC-3'	55.2	HQ266593
Seg-4	1730	OC1	554	62.53	18-1682	17	51	5'-GUAAAA	AAGAUAC-3'	56.4	HQ266594
Seg-5	1719	Tup	529	60.03	40-1629	39	93	5'-GUAAAA	AAGAUAC-3'	58.9	HQ266595
Seg-6	1668	OC2	537	59.44	23-1636	22	34	5'-GUAAAA	AGGUUAC-3'	56.5	HQ266596
Seg-7	1197	ViP	368	40.93	46-1152	45	48	5'-GUAAAA	AAGAUAC-3'	56.4	HQ266597
Seg-8	1183	T13	357	39.5	19-1092	18	94	5'-GUAAAA	AAGUUAC-3'	57.7	HQ266598
Seg-9	1049	Hel	317	34.19	59-1012	58	40	5'-GUAAAA	AAGAUAC-3'	54.1	HQ266599
		NS4	151	17.62	285-740	284	312				
Seg-10	707	NS3	214	23.41	19-663	18	47	5'-GUAAAA	AGGAUAC-3'	55.4	HQ266600
		NS3a	208	22.78	37-660	36	47				
**Total**	**17875**						**Consensus**	**5'-GUAAAA**	**A^A^/_G_^G^/_C_^A^/_U_UAC-3'**	56.3	

* In NS3 ORF, 1st, 2nd and 4th codons encode methionine, therefore putative NS3a starts at nucleotide position 64. For the abbreviations of putative proteins refer to Table 3.

**Table 2:** List of recognized *Orbivirus* species and proposed new species with their coding assignments and available genome sequence data.

			Genome Segments/Putative Proteins Encoded																				Vectors
		Seg 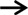	1	2		3			4			5		6	7			8		9		10	
Sl No	Serogroup/Species	Abbreviation	Pol	OC1	T2	OC1	T2	Cap	Cap	OC1	Tup	Cap	Tup	OC2	T13	ViP	Hel	T13	ViP	Hel	ViP	NS3	
1	*Bluetongue virus*	BTV	√	√	−	−	√	−	√	−	−	−	√	√	√	−	−	−	√	√	−	√	
2	*African horse sickness virus*	AHSV	√	√	−	−	√	−	√	−	−	−	√	√	−	−	√	√	−	−	√	√	
3	*Equine encephalosis virus*	EEV	√	−	√	√	−	−	√	−	−	−	√	√	−	√	−	√	−	√	−	√	
4	*Eubenangee virus*	EUBV	√	√	−	−	√	−	√	−	−	−	√	√	√	−	−	−	√	√	−	√	
5	*Epizootic haemorrhagic disease virus*	EHDV	√	√	−	−	√	−	√	−	−	−	√	√	−	√	−	√	−	√	−	√	CBOs
6	*Lebombo virus*	LEBV	√	−	√	√	−	−	√	−	−	−	√	√	√	−	−	−	√	√	−	√	
7	*Orungo virus*	ORUV	√	√		−	√	−	√	−	−	−	√	√	√	−	−	−	√	√	−	√	
8	*Palyam virus virus*	PALV	√	√	−	−	√	−	√	−	−	−	√	√	√	−	−	−	√	√	−	√	
9	*Warrego virus*	WARV	√	√	−	−	√	−	√	−	−	−	√	√	√	−	−	√	−	√	−	√	
10	*Wallal virus*	WALV	√	√	−	−	√	−	√	−	−	−	√	√	√	−	−	−	√	√	−	√	
11	*Changuinola virus*	CGLV	√	√	−	−	√	−	√	−	−	−	√	√	√	−	−	−	√	√	−	√	PBO
12	*Corriparta virus*	CORV	√	−	√	√	−	−	√	−	−	−	√	√	−	√	−	√	−	√	−	√	
13	*Ieri virus*	IERIV	−	−	−	−	−	−	−	−	−	−	−	−	−	−	−	−	−	−	−	−	
14	*Peruvian horse sickness virus*	PHSV	√	−	√	√	−	−	√	−	−	−	√	√	−	√	−	√	−	√	−	√	MBOs
15	*Umatilla virus*	UMAV	√	−	√	√	−	−	−	−	√	√	−	√	−	√	−	√	−	√	−	√	
16	*Wongorr virus*	WGRV	−	−	P*	−	−	−	−	−	−	−	−	−	−	−	−	−	−	−	−	−	
17	*Yunnan orbivirus*	YUOV	√	−	√	√	−	−	√	−	−	−	√	√	−	√	−	√	−	√	−	√	
18	*Chobar gorge virus*	CGV	√	−	√	−	−	√	−	√	−	−	√	√	√	−	−	−	√	√	−	√	
19	*Chenuda virus*	CNUV	√	−	√	−	−	√	−	√	−	−	−	√	−	√	−	√	−	√	−	√	TBOs
20	*Wad Medani virus*	WMV	√	−	√	−	−	√	−	√	−	−	√	√	−	√	−	√	−	√	−	√	
21	*Great island virus*	GIV	√	−	√		−	√		√	−	−	√	√	√	−	−	−	√	√	−	√	
22	*St'Croix river virus*	SCRV	√	−	√	√	−	−	√	−	−	−	√	√	−	√	−	√	−	√	−	√	TO
			**Genome Segments/Putative Proteins Encoded**																				**Vectors**
		**Seg** 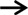	**1**	**2**		**3**			**4**			**5**		**6**	**7**			**8**		**9**		**10**	
**Sl No**	**Proposed species**	**Abbreviation**	**Pol**	**OC1**	**T2**	**OC1**	**T2**	**Cap**	**Cap**	**OC1**	**Tup**	**Cap**	**Tup**	**OC2**	**T13**	**ViP**	**Hel**	**T13**	**ViP**	**Hel**	**ViP**	**NS3**	
1	Pata virus	PATAV	√	√	−	−	√	−	√	−	−	−	√	√	−	√	−	√	−	√	−	√	CBO
2	Kemerovo virus	KEMV	√	−	√		−	√		√	−	−	√	√	−	√	−	√	−	√	−	√	TBO
3	Breu Branco virus		√	−	√	√	−	−	√	−	−	−	√	√	−	√	−	√	−	√	−	√	MBO
4	Sathuvachari virus	SVIV	√	−	√	√	−	−	√	−	−	−	√	√	−	√	−	√	−	√	−	√	MBO
5	Mobuck virus		√	−	√	√	−	−	√	−	−	−	√	√	−	√	−	√	−	√	−	√	MBO
6	Heramatsu virus	HERMV	√	√	−	−	√	−	√	−	−	−	√	√	√	−	−	−	√	√	−	√	CBO
7	Tibet orbivirus	TIBOV	√	√	−	−	√	−	√	−	−	−	√	√	√	−	−	−	√	√	−	√	CBO

For the abbreviations of putative proteins refer to Table 3. Prototype viruses of recognized species, for which full genomes are available, are highlighted in grey. Viruses sequenced in this study are highlighted in green. √ = Full length sequence are available; P* = Partial sequence only; CBO = *Culicoides*-borne orbivirus; MBO = Mosquito-borne orbivirus; TBO = Tick-borne orbivirus; TO = Tick orbivirus. Accession numbers for the sequences of each genome segment are provided in supplementary data Table S1.

**Table 3:** Coding assignments of the Tick-borne [Chenuda virus (CNUV), Chobar Gorge virus (CGV), Wad Medani virus (WMV), Great Island virus (GIV) and Kemerovo virus (KEMV)], *Culicoides-*borne [Bluetongue virus (BTV)], phlebotomine-borne [Changuinola virus (CGLV)] and mosquito-borne [Corriparta virus (CORV), Peruvian horse sickness virus (PHSV), Yunnan orbivirus (YUOV)] orbiviruses.

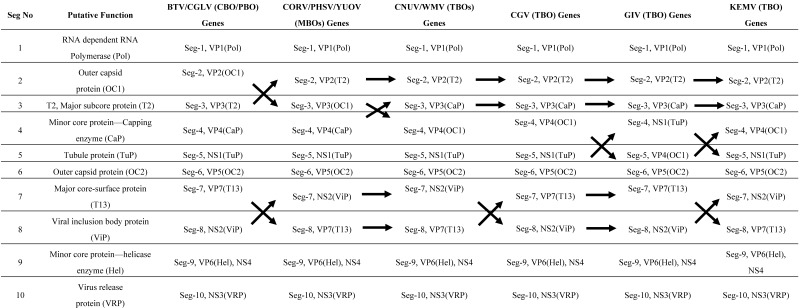

CBO = *Culicoides*-borne orbivirus; MBO = mosquito-borne orbivirus; TBO = Tick-borne orbivirus; TO = tick orbivirus. The arrows indicate the shift of corresponding segments in different *Orbivirus* species. Previous studies have indicated that BTV genome-segments 2, 3, 4, 5 and 6 are homologous to segments 5, 2, 3, 6 and 4, respectively, of GIV [13,32]. However, the analyses of TBOs presented here indicate that Seg-2, 3, 4 of BTV are homologous to Seg-4, 2 and 3 of the TBOs. The genome segments of the different orbiviruses are numbered in order of decreasing size. The black arrows indicate the relative positions of homologous segments, where their size order has changed.

### 2.2. Sequence Analyses of the Chenuda virus (CNUV), Chobar Gorge Virus (CGV) and Wad Medani Virus (WMV) Genome Segments

Full length nucleotide sequences for Seg-1 to Seg-10 of Chenuda virus (CNUV), Chobar Gorge (CGV) and Wad Medani virus (WMV) (ORC isolates: EGY1954/01 NEP1970/01 and SUD1952/01, respectively) have been determined and submitted to GenBank, with accession numbers KP268794 to KP268803; KP268784 to KP268793; and KP268804 to KP26813, respectively. The properties of the tick-borne orbiviruses genome segments and their encoded proteins are given in Table 1, allowing the coding assignments to be determined for each genome segment and compared to data for other orbiviruses (Table 2). The total genome of CNUV, CGV, WMV, GIV and KEMV are 17,910, 17,899, 18,009, 17,858 and 17,874 base pairs (bp), respectively. Although these viruses show some differences in the sizes of their equivalent genome segments, their full genome sizes are comparable, although smaller (~ 1 kb) (possibly reflecting their smaller OC1 protein and gene) than the insect-borne orbivirus (IBO) genomes, which range from 18,915 bp in Palyam virus (a CBO), to 19816 bp in Yunnan orbivirus (a MBO).

The average GC content of the *Culicoides*-borne orbiviruses genome segments is between 39% in Warrego virus (WARV) to 45.9% in equine encephalosis virus (EEV). Changuinola virus (CGLV), which is a phlebotomine-borne orbivirus, has 41.7% GC, while the mosquito-borne orbiviruses have a more diverse GC content between 36.7% in Peruvian horse sickness virus (PHSV) to 45.1% in Corriparta virus (CORV). In contrast, the genomes of all of tick-borne orbiviruses that have been sequenced have a markedly higher GC content than the insect-borne orbiviruses, between 53% (CGV) and 58.1% (GIV) (Table 1). St Croix River virus (SCRV), which is a tick-associated virus and must therefore also replicate in tick cells, also has a high GC content of 51.9%.

Like the other orbiviruses, all of the genome segments of the TBOs have conserved regions at their 5'and 3' ends, and the first and last two nucleotides in all segments are inverted complements (Table 1). The 5' terminal dinucleotides and 3' trinucleotides are also identical to those found in members of other *Orbivirus* species. Collectively, the terminal non-coding regions (NCR) represent 3.67%, 3.63%, 4.11%, 5% and 4.32% of the CNUV, CGV, WMV, GIV and KEMV genomes, respectively. Like most genome segments from other orbiviruses, RNAs of CNUV, CGV, WMV, GIV and KEMV all have shorter 5' than 3' NCRs (Table 1).

Coding assignments for the TBO genome segments are shown in Table 3. Most of the TBO RNA segments are monocistronic, containing a single major open reading frame (ORF), which spans almost the entire length of the positive strand. The coding assignments for CGV, CNUV and WMV are identical, except in Seg-7 and 8, which have swapped their relative migration order/size in CGV. The TBO coding assignments are different from those of the insect-borne orbiviruses (Table 3), primarily because of differences in the sizes of the OC1 gene, which is much smaller (approximately half the size of the homologous gene from the insect-borne orbiviruses).

As previously reported for BTV and Great Island virus (GIV) [18,19], Seg-9 of the TBOs also has two overlapping but out-of frame ORFs. The upstream ORF, which spans almost the entire length of Seg-9, encodes the viral helicase, VP6(Hel), while the second and overlapping +2 ORF, encodes NS4 (Table 1). NS4 is hydrophilic and exhibits a high level of variability in both length and sequence, between the members of different *Orbivirus* species, sharing aa identities that range between 3.7% (between CNUV and WARV) to 51.3% (between BTV-8w and EHDV-1w). NS4 of CGV, CNUV, WMV, GIV and KEMV is 238aa, 183aa, 189aa, 190aa and 151aa long, respectively. NS4 of CGV is approximately 20% larger than in the other TBOs and is larger even than the CGV NS3 protein. The insect-borne orbiviruses usually have a smaller NS4 (76 aa in EHDV to 152 aa in CORV), although this does not significantly affect the overall size of Seg-9, which also codes for the viral helicase, VP6.

### 2.3. Phylogenetic Analyses of the Tick-Borne Orbiviruses VP1/Pol Protein

**Figure 2:**
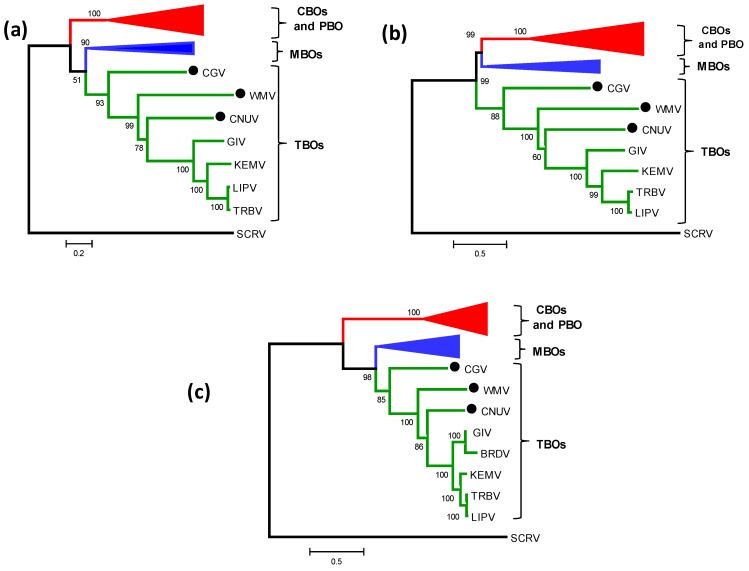
Maximum likelihood (ML) trees showing phylogenetic comparisons of (**a**) VP1 protein, (**b**) VP1 nucleotide and (**c**) T2 protein sequences of CNUV, CGV and WMV with other *Orbivirus* species. The numbers at nodes indicate bootstrap confidence values after 1000 replications. The scale bar represents the number of substitutions per site. The CNUV, CGV and WMV isolates characterized in this study are marked with a black dot. CGV = Chobar Gorge virus; CNUV = Chenuda virus; WMV = Wad Medani virus; CBOs = *Culioides*-borne orbiviruses; PBO = Phlebotomine-borne orbivirus; MBOs = Mosquito-borne orbiviruses; TBOs = Tick-borne orbiviruses. In phylogenetic trees, CBOs are depicted in red, MBOs are depicted in blue, TBOs are depicted in green and tick orbivirus is depicted in black. Full names of virus isolates and accession numbers of proteins used for comparative analysis are listed in Table S1 (supplementary data).

The orbivirus RNA dependent RNA polymerase (Pol) (encoded by Seg-1), is highly conserved and has previously been used in phylogenetic studies to classify viruses from the family *Reoviridae*, at both the species and genus level [11,22,26,33]. Phylogenetic comparisons of VP1(Pol)/Seg-1 showed higher sequence identity levels between the TBOs, than with the insect-borne orbiviruses (Table 4). Three groups were identified (Figure 2a,b) that correlate with the arthropod vectors used by each virus: one group consists of the CBOs and PBOs; a second group includes the MBOs; while the third group comprises TBOs. Distinct branching of CNUV, CGV, WMV and GIV within the TBO group again supports their classification within distinct *Orbivirus* species. In contrast, GIV and KEMV group more closely together, consistent with their current classification as different subgroups within the same *Orbivirus* species. As previously suggested [13], SCRV (which is a distant member of the genus that is thought to be a tick orbivirus (TO) rather than a TBO) ‘roots’ all other orbiviruses (Figure 2a,b).

**Table 4:** Percent amino acid and nucleotide identities of CNUV, CGV, WMV, GIV and KEMV viruses with other orbiviruses in VP1, T2 and T13 protein/genes.

	CNUV			CGV			WMV			GIV			KEMV		
	VP1	T2	T13	VP1	T2	T13	VP1	T2	T13	VP1	T2	T13	VP1	T2	T13
**% aa identities**															
BTV8w (CBO)	46.7	37.1	23.6	47.8	37.1	26.3	43.2	35.8	24.9	46.2	36.2	21.5	45.1	35.7	24.4
PHSV (MBO)	49.5	46.6	32.9	51.6	46.2	28.4	46.9	45.5	31.6	47.6	45.2	29.5	47.9	45.4	30.1
SCRV (TO)	39.5	24.9	16.5	40.3	24.7	18.9	39.2	24.6	22.3	41.5	24.6	17.1	39	25.1	18.5
TBOs															
CGV	55.3	50.2	34.5	----	----	----	----	----	----	----	----	----	----	----	----
WMV	54.2	59.1	47.5	51.9	47.7	34.6	----	----	----	----	----	----	----	----	----
GIV	58.3	62.4	52.8	53.7	51.5	35.3	54.2	57.9	52	----	----	----	----	----	----
KEMV	57.6	64.5	50.6	52.5	53.4	35.6	54.8	57.8	50.8	72.8	82.8	82.1	----	----	----
**% nt identities**															
BTV8w (CBO)	50.6	45.4	34.4	52.3	45.2	36.5	49.1	43.9	35.4	49.7	44.7	36.4	49.7	44.8	35.4
PHSV (MBO)	51.5	49.8	42.5	52.5	51.6	40.1	50.2	48.4	42.5	49.8	48.2	38.7	51.1	48.1	40.4
SCRV (TO)	46.1	38.4	34.3	46.7	38.9	33.4	46.5	38.5	36.3	47.8	38.6	34.2	46.1	39.1	35
TBOs															
CGV	53.9	53.7	43.5	----	----	----	----	----	----	----	----	----	----	----	----
WMV	54.6	57.8	51.5	53.1	53.2	45.4	----	----	----	----	----	----	----	----	----
GIV	56.5	60.6	54.3	54.2	54.1	46.1	55	58.2	56.2	----	----	----	----	----	----
KEMV	57.1	61.6	56.6	53.8	54.6	44	55.6	57.2	55.4	65.4	73.4	70.9	----	----	----

CBO = *Culicoides*-borne orbivirus; MBO = Mosquito-borne orbivirus; TBO = Tick-borne orbivirus; TO = Tick orbivirus.

### 2.4. Phylogenetic Relationships of the Tick-Borne Orbivirus Subcore-Shell ‘T2’ Protein

BlastX comparisons to homologous proteins from other orbiviruses, identified VP2 (encoded by Seg-2) of CNUV, CGV and WMV as the inner sub-core shell ‘T2’ protein. A phylogenetic tree constructed for orbivirus T2 proteins, separated the different isolates into groups that correlate with their different vectors, in a manner similar to the VP1 tree (Figure 2c). Three distinct clusters/groups were identified: one group, in which VP3(T2) is encoded by Seg-3, consisted of the CBOs and PBOs; while the second and third groups included MBOs and TBOs, respectively, in which VP2(T2) is encoded by Seg-2.

CNUV, CGV, WMV, GIV all branch separately in the Seg-2 and T2 protein trees, within TBO group, confirming that they represent distinct species. GIV and KEMV again group more closely together, consistent with their current classification as different subgroups within the same *Orbivirus* species (Figure 2c). SCRV again branches separately from the other orbiviruses. Pairwise aa/nt identities for T2 protein/gene were given in Table 4.

### 2.5. Phylogenetic Comparisons of the TBO Outer-Core T13 Protein

The most abundant orbivirus structural protein, VP7(T13), is a strongly immuno-dominant serogroup-specific antigen [34] and is highly conserved within each *Orbivirus* species. Phylogenetic trees (ML trees) constructed for the aa sequences of VP7(T13) (supplementary Figure S1a) exhibited a topology similar to the T2 and VP1(Pol) trees, with three distinct groups that correlate with the vectors used by each virus (the CBOs/PBO, MBOs and TBOs). T13/Seg-7 of CGV, CNUV, WMV showed highest identity levels with GIV (35.3/46.1%, 52.8/54.3%, 52/56.2% aa/nt, respectively), supporting their classification within distinct species. Although the TBOs consistently showed lower aa/nt identity levels with the insect-borne orbiviruses (<33%/42.5%), their relationships to the mosquito-borne orbiviruses are closer than to the *Culicoides-*borne or phlebotomine-borne orbiviruses (Table 4).

### 2.6. Phylogenetic Comparisons of Orbivirus Outer capsid Protein 1 (OC1)

Outer capsid protein one (OC1) determines *Orbivirus* serotype and is highly variable in both its aa sequence and size. OC1 is encoded by Seg-2 (VP2) in the PBO and CBOs (represented by BTV), by Seg-3 (VP3) in the MBOs (represented by PHSV) and by Seg-4 (VP4) in the TBOs [5,11,13]. OC1 of the TBOs is approximately half the size of the equivalent protein of the CBOs.

**Figure 3:**
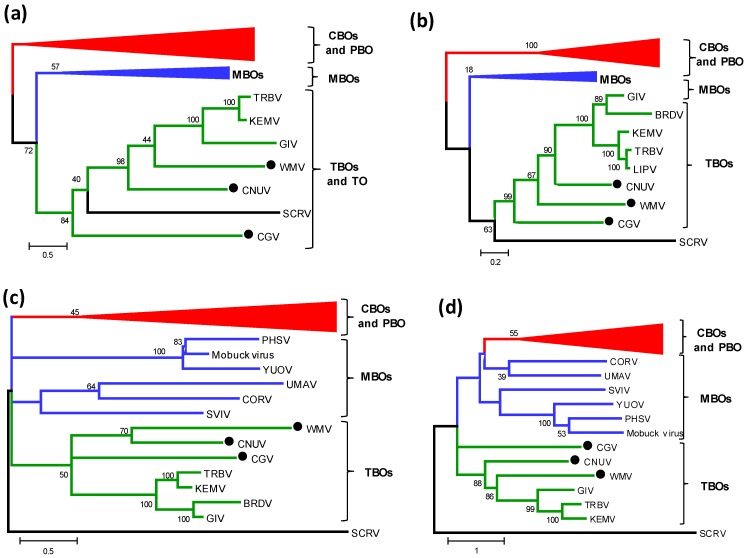
Maximum likelihood (ML) trees showing phylogenetic comparisons of amino acid sequences of (**a**) OC1 protein; (**b**) OC2 protein; (**c**) NS3 protein; and (**d**) VP6(Hel) protein of tick-borne orbiviruses with insect borne viruses. The numbers at nodes indicate bootstrap confidence values after 1000 replications. The scale bar represents the number of substitutions per site. The CNUV, CGV and WMV isolates characterized in this study are marked with a black dot. In phylogenetic trees, CBOs are depicted in red, MBOs are depicted in blue, TBOs are depicted in green and tick orbivirus is depicted in black color. Full names of virus isolates and accession numbers of proteins used for comparative analysis are listed in Table S1 (supplementary data). CGV = Chobar Gorge virus; CNUV = Chenuda virus; WMV = Wad Medani virus; CBO = *Culioides*-borne orbiviruses; MBOs = Mosquito-borne orbiviruses; TBOs = Tick-borne orbiviruses.

The aa sequence of the OC1 protein is more variable (within each *Orbivirus* species) than any of the other viral proteins, thought to reflect immune-selective-pressure from neutralizing antibodies (targeting OC1) that are generated by the vertebrate host [35,36]. However, despite this high level of serotype-specific variation in OC1, the ML tree constructed for this protein again showed three major clusters that correspond with the arthropod vectors used by each virus (like those for the Pol, T2 and T13 proteins) (Figure 3a). This consistent clustering, together with the higher sequence variation and major size differences observed in OC1, suggests that there is selective pressure to maintain the size and sequence (structure/function) of OC1 within each group.

### 2.7. Phylogenetic Analysis of Other Structural and Non-Structural Proteins of the Tick-Borne Orbiviruses

Phylogenetic trees constructed for the other structural and non-structural proteins of the TBOs show that the VP5 (OC2) (Figure 3b), VP3 (CaP), NS1(TuP) and NS2 (supplementary Figure S1b–d) all show similar relationships to those seen in VP1(Pol), sub-core ‘T2’ and core surface ‘T13’ proteins, with distinct monophyletic groups for the TBOs, MBOs and CBOs/PBO. Although NS3 (Figure 3c) and VP6(Hel) (Figure 3d) of the MBOs also cluster together in the phylogenetic trees, again grouping according to their vectors, both proteins (from the MBOs) form two subgroups. Although insufficient data is available concerning which mosquito species transmits each of these viruses, this sub-grouping suggests that they might use different groups or species of mosquito as vectors.

In general, the phylogenetic trees for all of the orbivirus proteins indicate that members of each virus species group closely together, while members of distinct species are branched separately, regardless of the protein selected.

This reflects a relatively high level of conservation between homologous segments and proteins within each *Orbivirus* species, likely reflecting important functional and/or structural interactions and constraints on each of the RNAs and proteins. These functional interactions may restrict genome segment exchange/reassortment, to viruses within the same *Orbivirus* species, and suggests that any novel orbivirus isolate would be identifiable (at the virus-species level) based on a phylogenetic analysis of any of its proteins/genes.

## 3. Discussion

Different orbivirus serogroups/species were originally identified and distinguished by a combination of their biological origins (host and vector), clinical signs and group-specific serological assays, including complement fixation (CF) and agar-gel immuno-precipitation (AGIP) tests and more recently serogroup-specific ELISA. However, significant similarities exist in the host ranges, clinical signs, arthropod vectors, distribution and serological properties between members of some different *Orbivirus* species. These similarities can result in low level or ‘one-way’ cross-reactions in serological assays (e.g., between bluetongue viruses (BTV); Epizootic hemorrhagic disease viruse*s* (EHDV); and Eubenangee viruses (EUBV)), making virus detection and conclusive identification more difficult, particularly if the viruses can co-circulate and can cause mixed infections [1,37,38,39,40]. Reliable detection, identification and differentiation of different orbiviruses, using conventional serological methods are also labor intensive and are hampered by the limited availability of reference virus strains and antisera for representatives of all different *Orbivirus* species/serogroups.

Electrophoretic analysis of orbivirus genome segments on 1% agarose gels (AGE) usually shows highly conserved size distributions and consequently migration patterns (electropherotype) within individual serogroups/species [1,41]. However, deletion, insertion or concatemerization events can occur that can cause significant changes in the migration of individual segments and the electropherotype of virus strains within a single species, as seen within the EHDV serogroup/species [42].

Differences in the size of equivalent genome segments between the CBOs, MBOs and TBOs, have been previously reported by several authors [5,11,26,32,43]. However, the migration patterns of different TBO species show significant similarities, exhibiting a 2-4-4 pattern that is distinct from those of the CBOs and MBOs (3-3-4 or 2-4-4 pattern).

With the advent of more rapid and reliable sequencing methods, full genome sequence data have been generated for reference strains of many *Orbivirus* species [5,11,26,28,30,41,44,45]. The resulting sequence data sets which can be easily accessed for phylogenetic comparisons, now represent a primary tool for identification and classification of novel orbivirus isolates [22,26,27,33,43]. Such comparative studies also enhance our understanding of virus evolution and strain movements (molecular epidemiology).

The intra-species genealogical and phylogenetic relationships of the CBOs (BTV, EHDV and AHSV) have been extensively studied, based on all ten genome segments/proteins [28,41,46,47,48,49,50]. In contrast, the wider inter-species relationships of different orbiviruses have only been studied for some of the more conserved proteins (e.g., VP1(Pol) and T2 proteins) and only for a limited number of species [5,11,13,14,22,26,45]. Since the choice of genomic region and the length of the sequences analyzed could affect phylogenetic inferences [51], we have analyzed full genome sequences’ for representative isolates (CNUV, CGV and WMV) of the TBOs, providing ‘reference data-sets’ for species identification. These sequences were also compared to previously published data for other tick-borne orbiviruses: GIV and KEMV.

The orbivirus polymerase ‘Pol’, sub-core-shell ‘T2’ and outer-core ‘T13’ proteins are all highly conserved. They have intra-species identity levels of >73%, >83%, and >73% aa identity (in BTV and EHDV) and maximum inter-species aa identity levels of 73%, 80% and 66%, respectively, between closely related virus species (such as BTV and EHDV) [5,11,46,49]. These genes/proteins have previously been used as ‘markers’ for identification and classification of both existing and novel orbivirus isolates [5,7,11,27,33,49]. They have also provided targets for development of *Orbivirus* species and genus-specific RT-PCR assays for virus typing, diagnosis and virus discovery [8,11,25,52,53,54].

The tick-borne orbiviruses analyzed here, CNUV, CGV and WMV, share less than 65% aa identity in all three conserved proteins Pol, T2 and T13 confirming their classification as distinct species within the genus *Orbivirus*. However GIV and KEMV share 72.8%, 82.8% and 82.1% aa identity in Pol, T2 and T13 proteins, respectively, very close to, or just beyond the previous maximum levels of variation detected within the CBO species (BTV and EHDV). It was therefore proposed that these two viruses could be recognized as two distinct species [13,14]. However, one of the primary determinants of virus species within the family *Reoviridae* is the ability of the different viruses within the same species to exchange/reassort genome segments during co-infection of the same cell, leading to the production of viable progeny reassortant virus strains [1]. The compatibility of individual viruses for reassortment depends on the ability of their different proteins/RNAs to interact and function efficiently during transmission/replication and will therefore require compatible structures and sequences, providing a relevant measure of similarity. It has previously been reported that GIV and KEMV virus can reassort their genome segments under laboratory conditions [55] and they are therefore classified within different sub-groups of the same *Orbivirus* species. Further sequence analyses of other virus isolates from the *Great Island virus* species may identify ‘intermediates’ between the different strains already analyzed, potentially filling in gaps, and confirming their inclusion within a single virus species.

Phylogenetic comparisons of most orbivirus proteins (VP1(Pol), T2, T13, CaP, OC1, OC2, NS1 and NS2) show three ‘clusters’ that correspond to the arthropod vectors that transmit each virus (Figure 2 and Figure 3). These data and comparisons to the phylogenetic trees for different arthropod species [45], support the hypothesis that the orbiviruses have evolved through ‘*co-speciation*’ with their arthropod vectors and that the TBOs provide an ancestral ‘root’ for the insect transmitted orbiviruses [11,13,45]. Phylogenetic trees for the different proteins of the TBOs and MBOs show that they form two distinct phylogenetic clusters. For proteins VP1, T2, T13 NS1, NS2, OC1 and OC2 these groups originate from a common branch (Figure 2 and Figure 3) and are more closely related to each other than to the equivalent proteins of the CBOs. In contrast, the groups containing sequences of CaP, Hel, and NS3, of the MBOs cluster more closely with the CBOs than with the TBOs (Figure 3c,d and supplementary Figure S1b). The monophyletic grouping of the individual orbivirus proteins (each according to the vectors used by the virus) demonstrates that aa sequence identity levels in individual viral proteins are related to the group of vectors used for transmission. This suggests that the sequences and therefore the functionality of the different proteins may help to determine the vectors that can be used by each virus.

Some of the differences/heterogeneity in the genome segments and their order of migration, of the CBOs/PBO, MBOs and TBOs are caused by large variations in the relative size of the highly variable outer capsid protein OC1. This heterogeneity is due to acquired point mutations, insertions and deletions, as well as inter- and intra-genic recombination and gene duplications (concatemerization) over a long time periods [42,45].

In *Culicoides*-borne orbiviruses, OC1 is the second largest viral protein (VP2—Encoded by Seg-2: 110–120 kDa), while in the mosquito-borne orbiviruses it is slightly smaller (~10% smaller) (VP3—encoded by Seg-3: 90–100 kDa) and is smallest in tick-borne orbiviruses (~50% smaller) (VP4 encoded by Seg-4: 62–66 kDa) [45]. There are sequence similarities that provide evidence of multiple gene duplications events in the outer capsid proteins of EHDV [42]. It is considered likely that the large OC1 of the insect-borne orbiviruses is the result of a full length gene duplication (concatemerization) event of an ancestral TBO genome segment, followed by point mutations over time that have obscured the full extent of the repeated sequence.

Assuming that the tick associated orbiviruses (TBOs and TO) are ancestors of all other orbiviruses, duplication events may have led to the evolution of larger viral genes and proteins in the other groups [13,45]. Concatemerization, which may be a common feature during orbivirus replication, but usually remains unfixed in the virus population [42], however gene duplication could provide an important mechanism by which sequence variation and coding capacity is created over time. Interestingly, the TBOs have smaller genomes (at least by 1 kb) and higher GC content than the insect-borne orbiviruses, but have larger NS4 proteins (more than 183 aa) compared to the insect-borne orbiviruses (less than 152 aa).

Sequencing and phylogenetic analyses of virus genomes, provides a basis for classification, diagnosis and vaccine development and helps to identify recombinant/reassorted strains. This suggests that full genome sequencing will become an accepted standard for future molecular epidemiological studies. It will therefore be important to generate a full genome sequence database that includes representative members of all *Orbivirus* species. The full genome sequence reported here for reference strains of *Chenuda virus*, *Chobar Gorge virus* and *Wad Medani virus*, together with the earlier data for GIV and KEMV completes a genome data set for reference strains of the tick-borne *Orbivirus* species. This will not only help to identify novel tick-borne orbiviruses, but will also provide a useful tool for identification and study of other orbiviruses.

Full genome sequences are now available for reference strains of twenty of the twenty-two *Orbivirus* species recognized by ICTV. These data have provided a basis for proposals to ICTV to recognize seven novel *Orbivirus* species, the development, and testing (*in silico*) of relevant diagnostic assays, and provide support for molecular epidemiology/evolutionary studies to enhance our understanding of orbivirus diseases in vertebrates.

## 4. Materials and Methods

### 4.1. Viruses

The viruses used in this study EGY1954/01 (CNUV), NEP1970/01 (CGV) and SUD1952/01 (WMV) were obtained from the Orbivirus Reference Collection (ORC) at The Pirbright Institute. These viruses were originally taken from naturally infected animals by qualified veterinarians, as part of normal diagnostic testing procedures in the respective countries. CNUV and CGV were propagated in BHK-21 cells (clone 13 obtained from European Collection of Animal cell Cultures (ECACC—84100501), while WMV was grown in BSR cells (a clone of BHK) [56] or BHK cells, in Dulbecco’s minimum essential medium (DMEM) supplemented with antibiotics (100 units/mL penicillin and 100 μg/mL streptomycin) and 2 mM glutamine. Infected cell cultures were incubated until they showed widespread (100%) cytopathic effects (CPE). Viruses were harvested, aliquoted and used for dsRNA extraction, or stored in the orbivirus reference collection (ORC) at −80 °C.

### 4.2. Preparation of Viral dsRNA

Guanidinium isothiocyanate extraction procedure described by Attoui *et al.* [57] was used to extract intact genomic dsRNA from CNUV, CGV and WMV infected cell cultures. Briefly, the infected cell pellet was lysed in 1 mL of TRIZOL^®^ reagent (Invitrogen), mixed with 0.2 volume of chloroform vortexing and the mixture was incubated on ice for 10 min. Total RNA present in supernatant was separated from cellular debris and DNA by centrifuging at 10,000× *g* for 10 min at 4 °C. Removed single stranded RNA (ssRNA) by 2M LiCl precipitation at 4 °C overnight, followed by centrifugation at 10,000× *g* for 5 min. Equal volume of isopropanol and 750 mM ammonium acetate was added to supernatant and then viral dsRNA was allowed to precipitate for a minimum of 2 h at −20°C. The dsRNA was pelleted by centrifugation at 10,000× *g* for 10 min, washed with 70% ethanol, air dried and dissolved in nuclease free water (NFW). The RNA was either used immediately or stored at −20°C.

### 4.3. Reverse Transcription of dsRNA and PCR Amplification of cDNAs

The genome segments of CNUV, CGV and WMV were reverse-transcribed using a ‘full-length amplification of cDNA’ (FLAC) technique described by Maan *et al.* [44]. Briefly, a 35 base self-priming oligonucleotide ‘anchor-primer’, with a phosphorylated 5' terminus, was ligated to the 3' ends of the viral dsRNAs using the T4 RNA ligase, followed by reverse transcription using RT system (Promega). The resulting cDNAs were amplified using complementary primers to the anchor primer and the amplicons were analyzed by 1% agarose gel electrophoresis. For cloning purposes, a high fidelity KOD polymerase enzyme (Novagen) was used in the PCR.

### 4.4. Cloning and Sequencing of cDNAs

Purified amplicons of CNUV, CGV and WMV were cloned into the ‘pCR^®^-Blunt’ vector supplied with the Zero Blunt^®^ PCR Cloning Kit (Invitrogen). Recombinant plasmid vectors containing inserts were transformed into One Shot^®^ TOP10 competent cells, supplied with the cloning kit. Clones containing relevant inserts were identified by colony PCR using M13 universal primers. Plasmids were extracted from the clones identified using the QIAprep Spin MiniPrep Kit (Qiagen). The plasmids and PCR products were sequenced using an automated ABI 3730 DNA sequencer (Applied Biosystems).

### 4.5. Sequence Analysis and Phylogenetic Tree Construction

‘Raw’ ABI sequence data were assembled into ‘contigs’ using the SeqManII sequence analysis package (DNAstar version 5.0). The ORFs of CNUV, CGV and WMV were identified and translated into aa sequences for further analysis using EditSeq (DNAstar version 5.0). The putative function of each protein was identified by BlastX comparisons to homologous orbivirus (BTV) proteins in GenBank [58]. Multiple alignments of consensus sequences were performed using ClustalX (Version 2.0) [59], Clustal Omega [60] and MAFFT [61] to ensure proper alignment. Aligned protein sequences were back translated to nucleotide sequences using DAMBE [62]) or RevTrans 1.4 server available online [63] for further nucleotide analysis. The best fit amino acid (aa) and nucleotide (nt) models for Maximum likelihood (ML) analysis were determined using ProtTest 3.0 and jModeltest, respectively [64,65]. The models were also determined using MEGA 5 software. The consensus or simplest model given by Akaike information criterion (AIC) and Bayesian Information Criterion (BIC) was selected for ML tree construction. The nt model GTR (I+G) with 1000 bootstraps was used for construction of Seg-1 phylogenetic tree. The aa model rtREV (I+G+F) was used for ML phylogenetic construction of all orbivirus proteins except for OC1 and NS3 for which WAG (I+G+F) and JTT (I+G+F) models, respectively, were used. All phylogenetic trees constructions and pairwise distance calculations using p-distance parameter were performed using MEGA 5 [66,67]. GenBank nucleotide accession numbers for the sequences used for analysis and phylogenetic studies are listed in the Table S1 (supplementary data).

## Supplementary Files

Supplementary File 1
