# Supplementary material for: Genetic Characterization of the Tick-Borne Orbiviruses
Source: Viruses. 2015 Apr 28;7(5):2185–209. doi: 10.3390/v7052185 (PMC4452902; doi:10.3390/v7052185)
Supplement: Supplementary File 1 [file viruses-07-02185-s001.pdf]

Supplementary data

Table S1: Nucleotide accession numbers for sequences used in phylogenetic analysis.

Species	Virus (Isolate)	VP1	OC1	T2	CaP	NS1	OC2	T13	NS2	VP6	NS3
Bluetongue virus	**BTV-1e (IND1992/01)**	JQ282770	AJ585111	JQ282771	JQ282772	JQ282773	AJ586659	JQ282774	JQ282775	JQ282776	JQ282777
	**BTV-8w (NET2006/04)**	AM498051	AM498052	AM498053	AM498054	AM498055	AM498056	AM498057	AM498058	AM498059	AM498060
	**BTV-25**	GQ982522	EU839840	GQ982523	GQ982524	EU839841	EU839842	EU839843	EU839844	EU839845	EU839846
	**BTV-26 (KUW2010/02)**	JN255156	HM590642	HM590643	JN255157	JN255158	JN255159	HM590644	JN255160	JN255161	JN255162
African horse sickeness virus	**AHSV-1 (HS29/62)**	FJ183364	FJ183365	FJ183366	FJ183367	FJ183368	FJ183369	FJ183370	FJ183371	FJ183372	FJ183373
Epizootic haemorrhagic disease virus	**EHDV-1w (USA1955/01)**	AM744977	AM744978	AM744979	AM744980	AM744981	AM744982	AM744983	AM744984	AM744985	AM744986
	**EHDV-2e (JAP1959/01)**	AM745077	AM745078	AM745079	AM745080	AM745081	AM745082	AM745083	AM745084	AM745085	AM745086
Changuinola virus	**CGLV (BE AR 490492)**	JQ610655	JQ610656	JQ610657	JQ610658	JQ610659	JQ610660	JQ610661	JQ610662	JQ610663	JQ610664
Equine encephalosis virus	**EEV (HS103/06)**	FJ183384	FJ183385	FJ183386	FJ183387	FJ183388	FJ183389	FJ183391	FJ183390	FJ183392	FJ183393
Orungo virus	**ORUV (UGMP 359)**	JQ610675	JQ610677	JQ610676	JQ610678	JQ610679	JQ610680	JQ610681	JQ610682	JQ610683	JQ610684
Lebombo virus	**LEBV (SAAR 3896)**	JQ610665	JQ610667	JQ610666	JQ610668	JQ610669	JQ610670	JQ610671	JQ610672	JQ610673	JQ610674
Eubenangee viurs	**EUBV (AUS1963/01)**	JQ070376	JQ070377	JQ070378<br>AF530087*	JQ070379	JQ070380	JQ070381	JQ070382	JQ070383	JQ070384	JQ070385
	**TILV (AUS1978/03)**	JQ070366	JQ070367	JQ070368	JQ070369	JQ070370	JQ070371	JQ070372	JQ070373	JQ070374	JQ070375
Palyam virus	**CHUV**	AB018086	AB014725	AB014728	AB018087	AB018089	AB014726	AB014727	AB018090	AB018088	AB018091
	**DAGV**	---		AF530085	---	---	---	---	---	---	---

	**(B8112)**										
Umatilla virus	**UMAV (USA1969/01)**	HQ842619	HQ842621	HQ842620	HQ842623	HQ842622	HQ842624	HQ842626	HQ842625	HQ842627	HQ842628
	**SLOV**	NC_012754	---	NC_012755	---	---	---	---	---	---	---
Peruvian horse sickness virus	**PHSV**	DQ248057	DQ248059	DQ248058	DQ248060	DQ248064	DQ248061	DQ248063	DQ248065	DQ248062	DQ248066
Corriparta virus	**CORV**	KC853042	KC853044	KC853043, AF530086*	KC853045	KC853046	KC853047	KC853049	KC853048	KC853050	KC853051
	**CMPV**	---	---	EU789391*	EU789392*	---	EU789393*	EU789394*	---	EU789395*	---
Yunnan orbivirus	**YUOV**	AY701509	AY701511	AY701510	AY701512	AY701513	AY701514	AY701516	AY701515	AY701517	AY701518
	**MPOV**	---	EF591621	EF591620	---	---	---	---	---	---	---
Great Island virus	**GIV (CanAr 42)**	HM543465	HM543469	HM543466	HM543467	HM543468	HM543470	HM543471	HM543472	HM543473	HM543474
	**BRDV**	---	---	M87875	---	X82599	M58030	M87876	---	---	M83197
	**LIPVh (CzArLip 91)**	HM543475	---	HM543476	---	---	HM543477	---	---	---	---
	**TRBVh**	HM543478	---	HM543479	---	---	HM543480	---	---	---	---
	**KEMVh (EgAn 1169-61)**	HM543481	---	HM543482	---	---	HM543483	---	---	---	---
	**TRBV**	HQ266581	HQ266585	HQ266582	HQ266583	HQ266584	HQ266586	HQ266588	HQ266587	HQ266589	HQ266590
	**KEMV**	HQ266591	HQ266594	HQ266592	HQ266593	HQ266595	HQ266596	HQ266598	HQ266597	HQ266599	HQ266600
St Croix river virus	**SCRV**	NC_005997	NC_005998	NC_005999	NC_006000	NC_006002	NC_006001	NC_006004	NC_006003	NC_006005	NC_006006
Warrego virus	**WARV (AUS1969/01)**	KJ495755	KJ495756	KJ495757	KJ495758	KJ495759	KJ495760	KJ495761	KJ495762	KJ495763	KJ495764
Wallal virus	**WALV (AUS1978/01)**	KJ495745	KJ495746	KJ495747	KJ495748	KJ495749	KJ495750	KJ495751	KJ495752	KJ495753	KJ495754
	**MUDV (AUS1982/03)**	KJ495765	KJ495766	KJ495767	KJ495768	KJ495769	KJ495770	KJ495771	KJ495772	KJ495773	KJ495774
Wongorr virus	**WGRV (V5080, V195, V199, mrm13443)**	---	---	U56989*, U56990*, U56991*, U56992*	---	---	---	---	---	---	---
	**PARV**	---	---	U56993*	---	---	---	---	---	---	---

	**PIAV**	---	---	U56994[*]	---	---	---	---	---	---	---
**Pata virus**	**PATAV (CAF1968/01)**	JQ070386	JQ070387	JQ070388	JQ070389	JQ070390	JQ070391	JQ070393	JQ070392	JQ070394	JQ070395
**California mosquito pool virus**	**CMPV**	---	---	EU789391[*]	EU789392[*]	---	EU789393[*]	EU789394[*]	---	EU789395[*]	---
**Sathuvachari virus**	**SVIV (IAn66411)**	KC432629	KC432630	KC432631	KC432632	KC432636	KC432633	KC432635	KC432637	KC432634	KC432638
**Heramatsu virus**	**(KY-663)**	KC669539	KC66941	KC66942	KC66943	KC66944	KC66945	KC66946	KC66947	KC66948	KC66940
**Mobuck virus**	**(12-2)**	KF296322	KF296323	KF296324	KF296325	KF296329	KF296326	KF296328	KF296330	KF296327	KF296331

[*]only partial sequences are available. Pata virus (represented in grey colour) represents novel species in the genus *Orbivirus* (Belaganahalli et al., 2012). **Pol**=Polymerase, **OC1**=Outer capsid protein 1 (VP2 of BTV), **T2**=Inner core protein (T2 symmetry), **Cap**= Capping enzyme, **Tup**=Tubule forming protein or Tubular protein (NS1), **OC2**=Outer capsid protein 2 (VP5 of BTV), **T13**=Outer core protein (T13 symmetry), **ViP**=Viral inclusion body protein (NS2), **Hel**=Helicase protein.

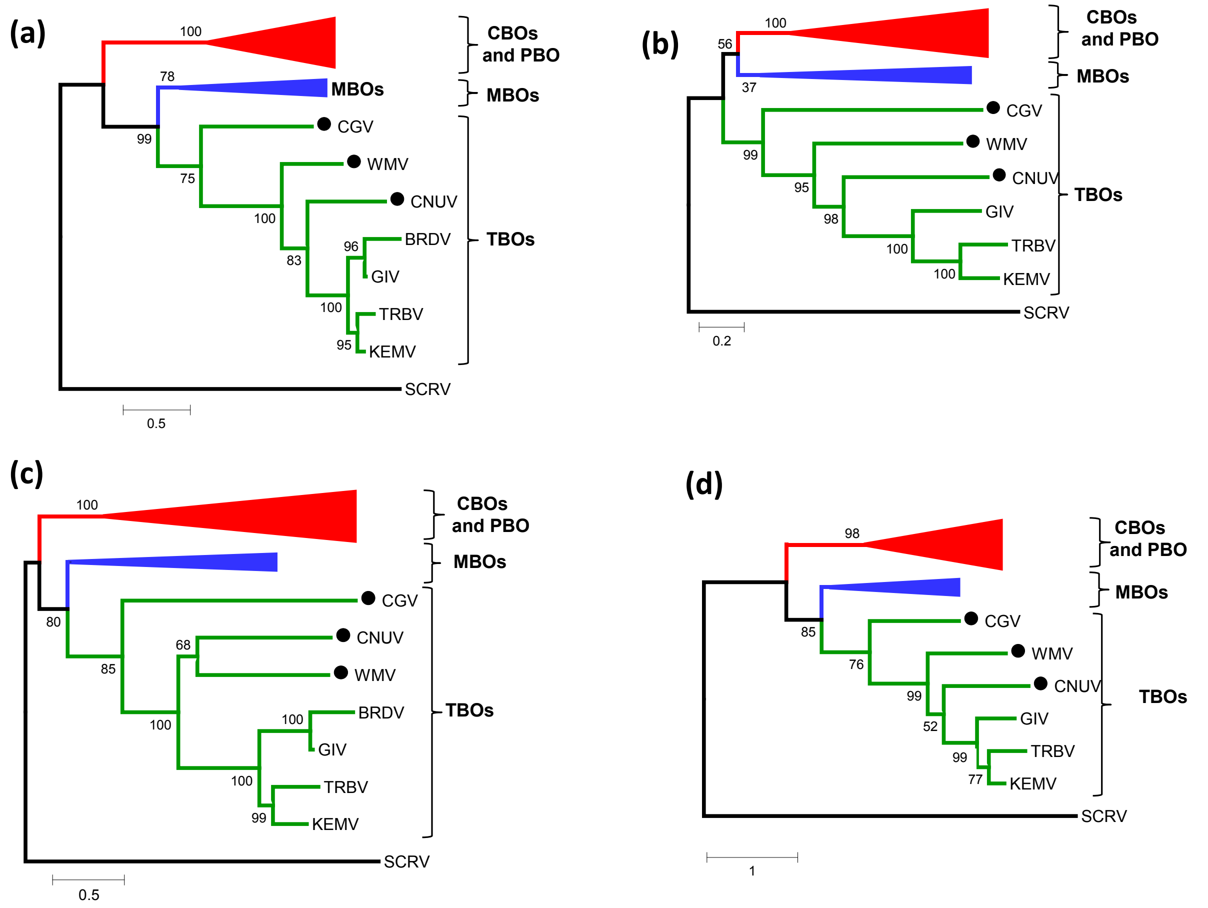

Figure S1: Maximum likelihood (ML) trees showing phylogenetic comparisons of amino acid sequences of (a) VP7(T13) protein, (b) VP3(CaP) protein, (c) NS1(TuP) protein and (d) NS2(ViP) protein of tick-borne orbiviruses with insect borne viruses.
The numbers at nodes indicate bootstrap confidence values after 1000 replications. The scale bar represents the number of substitutions per site. The CNUV, CGV and WMV isolates characterised in this study are marked with a black dot. In phylogenetic trees, CBOs are depicted in red, MBOs are depicted in blue, TBOs are depicted in green and tick orbivirus is depicted in black colour. Full names of virus isolates and accession numbers of proteins used for comparative analysis are listed in Table S1 (supplementary data). CGV = Chobar Gorge virus; CNUV = Chenuda virus; WMV = Wad Medani virus; CBO = *Culioides*-borne orbiviruses; MBOs = Mosquito-borne orbiviruses; TBOs = Tick-borne orbiviruses
